# Retro-orbital injection of FITC-dextran is an effective and economical method for observing mouse retinal vessels

**Published:** 2011-12-31

**Authors:** Shiqing Li, Tao Li, Yan Luo, Honghua Yu, Yuying Sun, Huanjiao Zhou, Xiaoling Liang, Juan Huang, Shibo Tang

**Affiliations:** State Key Laboratory of Ophthalmology, Zhongshan Ophthalmic Center, Sun Yat-sen University, Guangzhou, China

## Abstract

**Purpose:**

To assess the effects of retro-orbital (RO) injection of fluorescein isothiocyanate dextran (FITC-dextran) for observing mouse retinal vessels.

**Methods:**

Oxygen-induced retinopathy (OIR) was induced in 7-day postnatal (P7) C57BL/6J mice by exposing them to a 75% oxygen atmosphere for 5 days and then returning them to room air at P12. At P17, 45 P17 OIR mice and 12 normal mice received an RO injection of FITC-dextran. Five P17 OIR mice were perfused with FITC-dextran via the left ventricle. Retinal ﬂatmounts were viewed under a ﬂuorescence microscope and a confocal microscope. Following RO injection or left ventricular (LV) perfusion, the areas of neovascularization, as well as the total retina areas, were measured and analyzed using Image Pro-Plus 5.1 software.

**Results:**

The suitable volume of FITC-dextran for RO injection to observe the retinal vessels of a P17 OIR mouse was 0.05 ml, the 5% volume required to achieve similar results by administering FITC-dextran using LV perfusion. The total retinal vessels in P17 OIR mice could be fully observed under a ﬂuorescence microscope 3 s after RO injection of FITC-dextran. Our study showed that RO injection of FITC-dextran was suitable for observing the retinal vessels of all postnatal mice. There was no significant difference in evaluating retinal neovascularization between RO injection and LV perfusion of FITC-dextran (p>0.05).

**Conclusions:**

Retro-orbital injection of FITC-dextran is an economical method for observing retinal vessels in mice, when compared with LV perfusion of FITC-dextran.

## Introduction

During the physiologic development of the retina and the pathophysiological progression of retinal vessel–related diseases, such as retinopathy of prematurity (ROP), diabetic retinopathy, and retinal vessel occlusion, the changes in retinal vessels are observed to understand retinal vascularization. The retinal vascular abnormities of ROP can be studied in a well described oxygen-induced retinopathy (OIR) model [1]. Some substances, such as isolectin B4 [2], collagen IV [3], and fluorescein isothiocyanate dextran (FITC-dextran) [4], are used to observe retinal vessels. Studies show that high-molecular-weight FITC-dextran (2×10^6^) remaining in the vasculature without diffusion is a successful approach for observing mouse retinal vessels; in contrast, low-molecular-weight FITC-dextran (4×10^4^-5×10^4^) is not suitable as it leaks out of the vasculature [5]. The current method for sending FITC-dextran to mouse retinas is left ventricular (LV) perfusion [6,7]. The problem is that LV perfusion requires the researcher to perform many operations and these operations increase the mortality rate among the subject mice.

Previous studies [8,9] have shown that retro-orbital (RO) injection is a good and simple alternative for delivering low-molecular-weight substances such as viruses and radiotracers in vivo of mice. Researchers detected the injected materials later in organs such as the liver, lungs, and heart, proving that substances absorbed through the retro-orbital sinus could be widely distributed by the blood circulatory system. RO injection was also used for middle-molecular-weight fluorescein isothiocyanate BSA (7×10^4^) to observe liver tissues [10]. There are numerous anastomoses in the mouse orbital venous sinus [11], and the fenestrae of the mouse venous sinus is larger than the fenestrae of the microvascular wall [12]. Thus, we wondered whether high-molecular-weight FITC-dextran (2×10^6^) could also be sent with RO injection to the retinal vessels. In this study, we injected FITC-dextran into the orbital venous sinus of OIR and normal neonatal mice to evaluate whether such injection allowed us to fully observe the retinal vessels and to compare the effects of RO injection with the effects of LV perfusion.

## Methods

### Animals

All animal experiments adhered to the Association for Research in Vision and Ophthalmology (ARVO) statement for the Use of Animals in Ophthalmic and Vision Research and were approved by the Animal Care and Use Committee of the Zhongshan Ophthalmic Center, Sun Yat-sen University, Guangzhou, China.

C57BL/6J timed-pregnant mice were purchased from Southern Medical University, Guangzhou, China. The mice were housed in a specific pathogen-free animal facility at the Ophthalmic Animal Laboratory of the Zhongshan Ophthalmic Center. Feed and distilled water were available to the mice ad libitum, and fresh cages were provided on a weekly schedule.

### Oxygen-induced retinopathy model

The OIR mouse model was induced according to the protocol described previously [1]. Briefly, to induce retinal neovascularization, at postnatal day 7 (P7), mouse pups and their nursing dams were exposed to a 75%-oxygen atmosphere for 5 days and then returned to room air at P12. The retinal neovascularization of the OIR mice reached its peak at P17 [13]. The nursing dams were rotated from high oxygen to room air every 24 h to prevent oxygen toxicity.

### Retro-orbital injection of fluorescein isothiocyanate dextran

Before injection, FITC-dextran (FD2000S; Sigma-Aldrich, St. Louis, MO) was dissolved in ultrapure water at a concentration of 50 mg/ml, centrifuged at 10,000× g for 5 min, and collected as the supernatant. FITC-dextran was protected from light during the preparation.

P17 OIR mice, weighing 5.55–5.75 g, were anesthetized with an intraperitoneal (IP) injection of 10% chloral hydrate (2.5 ml/kg). The lateral canthus of the left orbit was chosen as the injection point. Gentle pressure with two fingers of the right hand was applied to the peri-orbital area to expose the mouse’s left eye. A 27-gauge needle with a 1 ml syringe attached gently pierced about 2–3 mm into the mouse’s orbital venous sinus with the bevel on the needle facing forward at a 45° angle [8], and then different volumes of FITC-dextran were injected.

To investigate the volume of injected FITC-dextran needed to observe the mouse retinal vessels, one of six different volumes of FITC-dextran (0.01 ml, 0.02 ml, 0.03 ml, 0.04 ml, 0.05 ml, or 0.10 ml) was injected into each of three P17 OIR mice, respectively, for a total of 18 P17 OIR mice injected during this step.

To investigate the optimal time for the enucleated eye, 27 P17 OIR mice eyes were enucleated at different elapsed time points after RO injection of 0.05 ml FITC-dextran (1 s, 2 s, 3 s, 5 s, 1 min, 5 min, 1 h, 2 h, and 5 h).

To evaluate whether the RO injection was suitable for a neonatal mouse, eyes from normal postnatal mice (n=12) were enucleated 5 min after RO injection of FITC-dextran at P0, P2, P8, and P17. The mice were injected with 0.02 ml, 0.02 ml, 0.04 ml, or 0.06 ml of FITC-dextran, respectively, depending on their weight (1.20 g, 1.74 g, 4.22 g, or 7.69 g).

### Left ventricular perfusion of fluorescein isothiocyanate dextran

Five P17 OIR mice, weighing 5.55 g–5.75 g, were anesthetized with an IP injection of 10% chloral hydrate. The thoracic cavity was opened to expose the pumping heart, and the right atrium was cut with scissors to interrupt the mouse’s blood circulatory flow, ensuring that FITC-dextran about to be injected in vivo into the left ventricle was not diluted by blood returning from the mouse’s lungs.

One ml of 50 mg/ml concentration of FITC-dextran was injected into the left ventricle. The mouse’s eyes were enucleated after a marked green coloration appeared on the front paws and tongue, indicating successful perfusion [6].

### Retinal flatmounts

Retinal ﬂatmounts were prepared according to the method described previously [14]. Briefly, the enucleated eyes were fixed in 4% paraformaldehyde for 40 min at room temperature. The cornea, iris, lens, and vitreous were gently removed under a stereomicroscope (Mz6; Leica, Wetzlar, Germany). Four to six radial incisions were made in the dissected retina, and it was flattened with a coverslip.

The retinal flatmounts were photographed at 50× original magnification with fluorescence microscopy (Zeiss Axioplan 2 imaging, Gottingen, Germany). The combined exposure time of the retinal flatmounts was 1.5 s. The superficial and deep vessels of the retina in each P17 OIR mouse were viewed under a confocal microscope (LSM 510 META; Carl Zeiss, Jena, Germany). The retinal segments were merged to generate an image of the total retina (Photoshop CS4; Adobe Systems Inc., San Jose, CA).

### Quantification of retinal neovascularization

To compare the success of injecting FITC-dextran to facilitate observation of retinal neovascularization (NV) using the RO injection method and the LV perfusion method, retinal flatmounts of three P17 mice in each group were used to quantify the area of retinal NV. Retinal flatmounts of P17 normal air mice (the control group) were also compared to those of the OIR mice (the OIR group). The retinal NV and the total retina were measured by outlining the corresponding areas and analyzed using Image Pro-Plus 5.1 software (Media Cybernetics Company, Silver Spring, MD), using the methods previously described [15].

### Statistical analysis

Statistical analysis was performed using SPSS software version 16.0 (SPSS, Inc., Chicago, IL). The data, showing the percentages of retinal neovascularization quantified by comparing the area of retinal neovascularization with the total area of the retina, were reported as the mean±standard deviation (SD) one-way ANOVA used for multiple-group comparison; an unpaired Student *t* test was used for the RO injection group and the LV perfusion group. A p value <0.05 was considered statistically significant.

## Results

### Retro-orbital injection of fluorescein isothiocyanate dextran in C57BL/6J mouse

**Figure 1:**
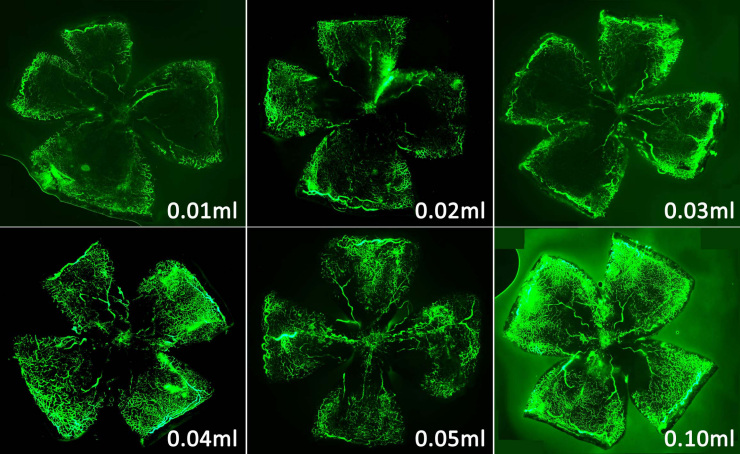
Representative retinal flatmounts from a P17 oxygen-induced retinopathy mouse after a retro-orbital injection of different volumes of fluorescein isothiocyanate dextran (FITC)-dextran. The fluorescence of 0.01–0.04 ml of FITC-dextran was too weak, while the fluorescence of 0.05 ml or 0.10 ml was fully observed. Original magnification: 50×.

The efficacy of RO injections varied depending on the amount of FITC-dextran injected. The RO injections of 0.01 ml, 0.02 ml, 0.03 ml, and 0.04 ml of FITC-dextran produced enhancements of small retinal vessels and neovascular tufts that were too weak to be fully shown with fluorescence, whereas RO injections of 0.05 ml and 0.10 ml of FITC-dextran enhanced those vessels and tufts sufficiently to be fully observed (Figure 1).

**Figure 2:**
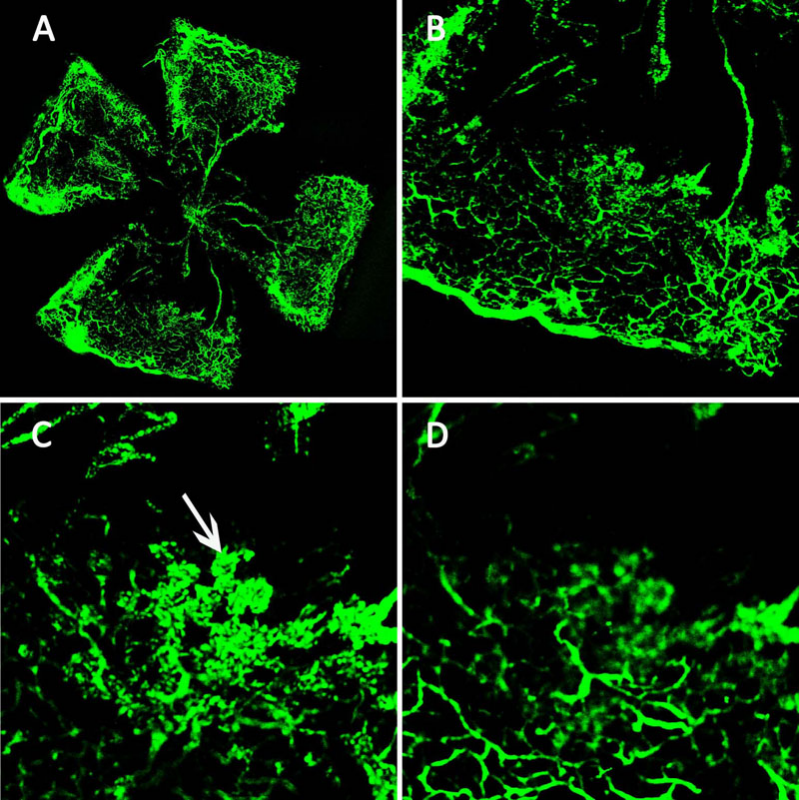
The superficial and deep vascular plexuses of the retina in a P17 oxygen-induced retinopathy mouse after a retro-orbital injection of fluorescein isothiocyanate dextran (FITC)-dextran. **A**: The retinal flatmount of a P17 oxygen-induced retinopathy mouse shows the fluorescence of small retinal vessels, avascular areas, and neovascular tufts. **B**: Original magnification: 50×. The superficial neovascular tufts (Arrow, **C**) and the deep vascular plexus (**D**) can be observed. Original magnification: 100×.

After the RO injection of 0.05 ml of FITC-dextran, the superficial and deep vascular plexuses of the retina were observed clearly under a confocal microscope; the structure of the neovascular tufts (arrow) was also distinguished (Figure 2). The superficial vascular plexus showed many neovascular tufts and large retinal vessels. The deep vascular plexus showed small retinal vessels.

**Figure 3:**
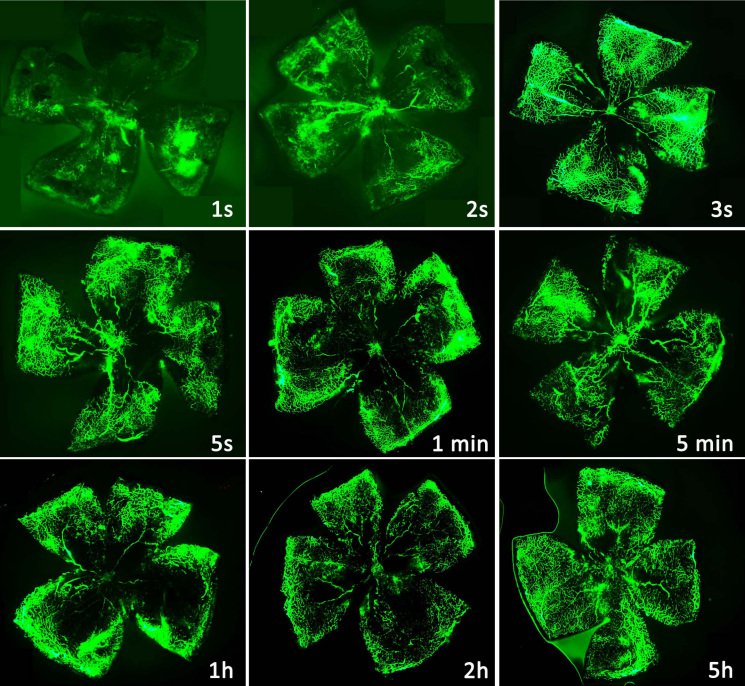
Time course of fluorescence of a P17 oxygen-induced retinopathy mouse after a retro-orbital injection of fluorescein isothiocyanate dextran (FITC)-dextran. The fluorescence of FITC-dextran in small retinal vessels was too weak at 1 s and 2 s, but the retinal vessels were fully observed at 3 s and could still be seen at 5 h. Original magnification: 50×.

The efficacy of the RO injections also varied with the time elapse between the injection and the observation. The fluorescence of small retinal vessels and neovascular tufts were too weak to be observed at 1 s and 2 s after the RO injection of 0.05 ml of FITC-dextran, but the small retinal vessels and neovascular tufts were fully observed from 3 s to 5 h after the RO injection (Figure 3).

**Figure 4:**
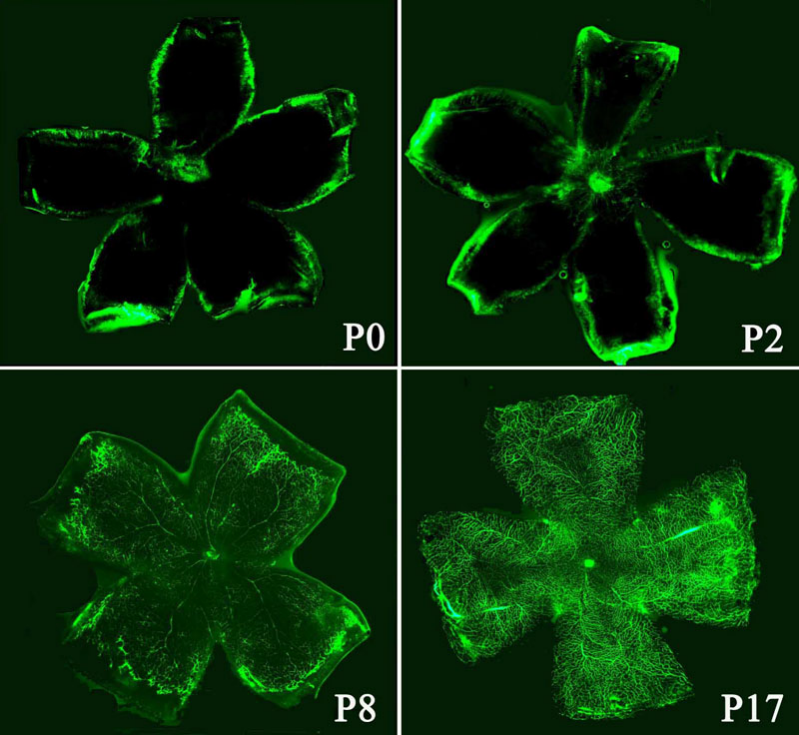
Retro-orbital injection of fluorescein isothiocyanate dextran (FITC)-dextran in newborn to P17 normal air mice. The superficial retinal vascular plexus grew outward from the optic nerve head (P0) to the peripheral tissue (P8), and the retinal vascular plexus of a P17 mouse was observed. Original magnification: 50×.

The mouse’s superficial retinal vascular structures grew outward radially after birth, from the central optic nerve to the retinal periphery. As shown in Figure 4, the fluorescence of the normal retinal vascular plexus was observed around the optic nerve in a P0 mouse and at the edge of the full retina in a P8 mouse. Figure 4 also shows the normal large and small retinal vessels of a P17 mouse.

### Comparison of the left ventricular perfusion and retro-orbital injection methods

The LV perfusion protocol usually called for injecting 1 ml of FITC-dextran concentrated to 50 mg/ml into each mouse heart, whereas the RO protocol injected only 0.05 ml per mouse.

Each LV perfusion surgical procedure took about 4 min and required four steps: providing anesthesia, opening the thoracic cavity, cutting the right atrium, and filling the left ventricle to initiate perfusion. The RO injection took about 30 s and required only two steps: anesthesia and injection into the orbital venous sinus.

The success rates of the RO and LV procedures were 100%. The time for the enucleated eyes for LV perfusion ranged from 1 min to 2 min, because the mouse died about 1 min after LV perfusion. However, the time for enucleated eyes for RO injection ranged from 3 s to 5 h. In our study, mouse recovery from general anesthesia was about 70 min, and no complications were found after RO injection of 0.05 ml of FITC-dextran.

**Figure 5:**
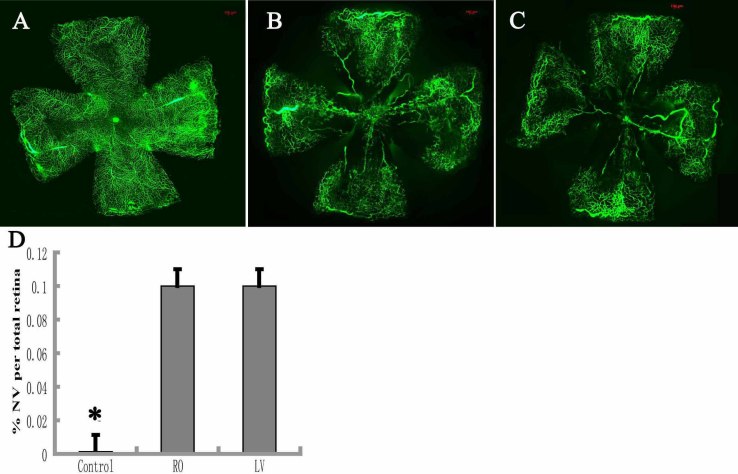
Quantification of the retinal area with neovascularization in P17 normal air mice and P17 oxygen-induced retinopathy (OIR) mice. The P17 normal air mice (control; **A**) and P17 OIR mice were given a retro-orbital injection (**B**) or left-ventricular perfusion (**C**) of fluorescein isothiocyanate dextran (FITC)-dextran. The neovascularization areas were outlined with Image Pro-Plus 5.1 software (Media Cybernetics Company, Silver Spring, MD). The percentage of the retinal neovascularization in the OIR group is a significant increase when compared to the control group (n=6), and no statistical difference was found between the retro-orbital injection (RO) group (n=6) and the LV group (n=6) **D**: Data shown are mean±standard deviation, *p<0.05. NV: neovascularization, LV, left-ventricular perfusion. Scale bar: 100 μm.

The percentage of retinal neovascularization in the control group (Figure 5A) and the OIR group (Figure 5B,C) was compared, and a significant increase in the OIR group was observed (F=0.007, p=0.001<0.05; Figure 5D). No significant difference in the percentage of retinal neovascularization was found between the RO group and the LV group (0.1030±0.0008 and 0.0997±0.0037, respectively; t=2.132, p=0.208 >0.05; Figure 5D).

## Discussion

Previous studies have shown that the recommended injection volume for an adult mouse is ≤0.15 ml [16], but no studies have established a safe injection volume for a P17 OIR mouse. Our study found that an RO injection of 0.05 ml of FITC-dextran satisfactorily facilitated the fluorescence of the superficial and deep vascular plexuses in a P17 OIR mouse, and the mice lived without accident; the optimal and safe volume for an RO injection of 50 mg/ml of FITC-dextran for P17 OIR mouse was 0.05 ml. Thus, we considered that the optimal and safe volume for an RO injection of 50 mg/ml of FITC-dextran for a mouse should be in accordance with the mouse weight and is about 0.009 ml/g (0.05 ml/5.55 g, the weight of P17 OIR mice).

A previous study indicated that the basal heart rate of a C57BL/6J mouse is more than 500 beats per min (bpm) [17], meaning that one complete circuit of blood takes less than 0.125 s. In our study, the fluorescence of flatmounted retinal vessels was not fully observed until 3 s after injection. This result indicates that 0.05 ml of FITC-dextran might not be fully absorbed to the mouse’s blood circulation system at 1 s and 2 s after the RO injection. The small retinal vessels and neovascular tufts were still seen at 5 h after the RO injection. Therefore, the recommended period for enucleating a mouse’s eye ranges from 3 s to 5 h after an RO injection.

FITC-dextran has been dissolved in PBS in some previous studies [1]. In our study, the FITC-dextran was dissolved in ultrapure water according to the product information for FITC-dextran provided by the manufacturer. Yardeni et al. [16] reported administering retro-orbital injections can reliably deliver volumes up to 0.01 ml in neonatal mice. The mouse retro-orbital sinus area is large with a substantial amount of blood flow [16], and the fenestrae of the orbital venous sinus is very large, which make a small volume (0.01 ml) of pure water be absorbed quickly (not more than 3 s) into the blood circulation of a P0 mouse. Moreover, there are potential spaces in the eye orbit for many fats. Thus, a small volume of pure water injected into the retro-orbit one time will not affect the retinal or retro orbital tissues very much compared with PBS (data not shown); especially in our study, the mice were sacrificed, and the eyes were enucleated 5 min after an RO injection of pure water. Therefore, using pure water to dissolve FITC-dextran did not affect the results in our study. However, when multiple RO injections are performed to obtain real-time retinal vessel images under a Micron II or III microscope without sacrificing the mice, PBS as an isotonic vehicle is a better choice than pure water, because multiple injections of pure water into the retro-orbit might cause some slight localized micro-cellular damages.

One study showed that an RO injection is suitable for a neonatal mouse [16], but no report has confirmed that the orbital venous sinus of a neonatal mouse is developed enough to absorb high-molecular-weight FITC-dextran. Figure 4 shows the fluorescence of retinal vessels in a P0 mouse, indicating that the fenestrae of the orbital venous sinus are large enough to absorb high-molecular-weight FITC-dextran. In addition, we observed the fluorescence of retinal vessels in P2, P8, and P17 normal mice. Therefore, RO injection of FITC-dextran is suitable for all postnatal mice. Previous studies also used many different types of injection needles, including 31-gauge or 32-gauge needles in newborn mice [9,18]. We found that a 27-gauge needle was suitable for newborn mice and all postnatal mice. As a 27-gauge needle is easy to find, RO injection of FITC-dextran does not require special equipment.

In our study, a 0.05 ml RO injection of FITC-dextran produced as good enhancement for observing retinal vessels as a 1 ml LV perfusion, because most of the FITC-dextran in a LV perfusion flows out of the right atrium after one rapid circuit through the mouse’s body. Compared with the LV perfusion method, the RO injection of the FITC-dextran for observing mouse retinal vessels has the following advantages: economical (an RO injection uses only 5% as much FITC-dextran as LV perfusion), simple (the RO injection procedure is easier than that of LV perfusion).

In summary, RO injection of high-molecular-weight FITC-dextran is suitable for all postnatal mice, and is an economical method for observing mouse retinal vessels, when compared with LV perfusion of FITC-dextran.
